# PFAS Pesticides: Contamination Pathways in Italy and the Need for Integrated Regulation

**DOI:** 10.3390/toxics14040325

**Published:** 2026-04-14

**Authors:** Emanuela Pace, Gianluca Maschio, Dania Esposito

**Affiliations:** Italian Institute for Environmental Protection and Research (ISPRA), 00144 Rome, Italy; gianluca.maschio@isprambiente.it (G.M.); dania.esposito@isprambiente.it (D.E.)

**Keywords:** PFAS, fate, risk assessment, pesticides, water contamination, OSOA, plant protection products, active substances, classification, persistence

## Abstract

In agriculture, the use of per- and polyfluoroalkyl substances (PFASs) as active substances in pesticides has increased over recent decades due to their chemical stability, their ability to alter cell membrane permeability, and their capacity to bind to target proteins. However, their intentional application to agricultural soils has led to progressive environmental accumulation. Their high persistence, mobility, and bioaccumulation potential, combined with documented toxicological effects, raise concerns for aquatic organisms and ecosystems. Monitoring surface and groundwater is essential to assess PFAS contamination. Data from the Italian monitoring plan show widespread contamination, despite the existing European regulatory framework designed to safeguard ecosystems and public health. The contamination is likely underestimated because monitoring programs currently target only a limited number of substances and PFAS metabolites and co-formulants are not included. Approximately 46 PFASs have been identified as active ingredients in pesticides, 29 of which are still authorized within the European Union, posing challenges for drinking water production and ecosystem protection. Existing regulatory regimes also differ in their evaluation procedures, which may lead to inconsistent conclusions regarding PFAS applications. Within the framework of the European “One Substance One Assessment” (OSOA) approach aimed at to ensuring the protection of human health and natural resources, this paper examines the properties of PFASs used as active substances in pesticides, their regulatory status, and their monitoring in Italy, highlighting the regulatory inconsistencies that result in the differential treatment of these substances compared with PFASs used in other sectors.

## 1. Introduction

Per- and polyfluoroalkyl substances (PFASs) are characterized by the presence of highly stable and chemically inert carbon–fluorine bonds, which confer unique physicochemical properties advantageous for numerous industrial and commercial applications. Consequently, PFASs are widely employed in products such as cosmetics, textiles, paints, pharmaceuticals, and pesticides. The production and daily use of these substances have led to their widespread environmental release, confirmed by numerous monitoring studies demonstrating their presence in water, air, soil, and food [1,2,3,4].

PFASs are often referred to as “forever chemicals” due to their high stability and near non-degradability, which makes them difficult to eliminate with conventional purification systems [5]. Their high persistence, mobility, and bioaccumulation potential, together with well-documented toxicological effects are the properties that cause concern and are making PFASs and/or their degradation products the subject of increasing international attention. In Northern Italy, a major contamination event, primarily driven by persistent emissions from a former chemical plant, was identified in 2013. Since then, the presence of PFASs in the environment and in humans has been the subject of extensive local investigation [6,7,8,9,10]. The widespread presence of PFASs in surface and groundwater bodies at the national level was demonstrated by a preliminary official survey [11,12]. Monitoring of Italian drinking water revealed the presence of PFASs in 79% of the samples analyzed, often as mixtures of multiple compounds [13]. A notable source of PFAS release into the environment is attributable to the use of various Plant Protection Products (PPPs) containing PFASs as active ingredient or as degradation by-product, which are applied directly on agricultural crops with potentially widespread dispersion. Analysis of Italian food products reveals increasing levels of PFAS contamination in fruit and vegetables [14]. Although pesticide monitoring in water has been conducted at the national level in Italy since 2003 [15], no studies have been conducted focusing on the contribution of PFAS pesticides to contamination.

To mitigate the substantial risks posed by PFASs to human health and the environment, the European Chemicals Agency (ECHA) is currently assessing a proposal for a so-called “universal PFAS” restriction aimed at drastically reducing their use [16,17]. An update to the Background Document of the restriction proposal was published in 2025, identifying additional use sectors (including technical textiles, mechanical applications, and other industrial uses) and evaluating alternatives to a total ban [18,19]. The final decision will be made by the European Commission after the Risk Assessment Committee and Socio-Economic Assessment Committee have delivered their opinions, which are expected to occur by the end of 2026.

In the EU, active substances in PPPs are regulated by an approval system under the respective regulation and therefore fall outside the scope of the PFAS restriction proposal. These existing regulatory regimes differ in their evaluation procedures, which may lead to inconsistent conclusions regarding PFAS applications. The universal restriction proposal covers all industrial uses of PFASs, considering alternatives and sectoral exemptions. In the approval of plant protection products, however, the evaluation is specific to each pesticide, and some PFAS substances can still be authorized if they meet the applicable regulatory criteria.

The main aim of this work is an analysis on the identification, properties, and regulatory status of PFASs used as active substances in PPPs (so-called “PFAS pesticides”), with specific regard to their environmental pressure and to surface and ground water monitoring in Italy.

## 2. Materials and Methods

### 2.1. Identity of the PFAS Pesticides

In this study, the identification of PFAS pesticides refers to the definition of PFASs used for the purpose of a restriction proposal on PFASs [18] that is based on the 2021 OECD definition [20] (with a few exceptions supposed to not fulfill the concern of high persistence) and is as follows:

“Any substance that contains at least one fully fluorinated methyl (CF_3_-) or methylene (-CF_2_-) carbon atom (without any H/Cl/Br/I attached to it).

A substance that only contains the following structural elements is excluded from the scope of the proposed restriction: CF_3_-X or X-CF_2_-X′, where X = -OR or -NRR′ and X′ = methyl (-CH_3_), methylene (-CH_2_), an aromatic group, a carbonyl group (-C(O)-), -OR″, -SR″ or –NR″R‴, and where R/R′/R″/R‴ is a hydrogen (-H), methyl (-CH_3_), methylene (-CH_2_-), an aromatic group or a carbonyl group (-C(O)-).”

The PFAS subgroups of active ingredients used in plant protection products typically contain one or more CF_3_-groups in their molecular structure, most often attached to aromatic rings, with the possible formation of metabolites and/or degradation products that are extremely stable and potentially hazardous. A non-exhaustive list of active substances for plant protection products covered by the current PFAS definition was provided in Annex A of the draft Background Document of the restriction proposal [19].

### 2.2. Statistics

Data on the sales of active substances in plant protection products were extracted from the database of the Italian National Institute of Statistics [21].

Data on pesticide monitoring were extracted from the Italian reports on pesticide monitoring in waters [15], prepared within the National System for Environmental Protection (SNPA). Water sampling and analytical measurements were carried out by Italian Regional Environmental Agencies under the Water Framework Directive (WFD—Directive 2000/60/EC) monitoring program. In particular, monitoring stations were selected across the country based on an analysis of the pressures and risks associated with the use of pesticides in agriculture: datasets from 2017 to 2021 were collected in this study and the monitoring data on the PFAS pesticides were extracted. The average number of samples analyzed for PFAS pesticides corresponds to 27,221 for surface water and 13,507 for groundwater; moreover, statistical analyses were derived from the average annual concentration of each monitored substance at each sampling station, as defined in the Italian reports on pesticides [15]. Because the focus of the statistical analyses is the occurrence of PFAS pesticides rather than their compliance with the regulatory threshold values, in this study, a substance was considered present in surface or groundwater stations if the average annual concentration was ≥LoQ (Limit of Quantification). As a general remark, it is noted that in the framework of the WFD, the contamination level in surface water refers to Environmental Quality Standard (EQS) established for 45 Priority Substances (Directive 2008/105/EC). Moreover, for some other river basin-specific pollutants, the EQS are defined at the national level (D.lgs. 172/2015). For all other pesticides (including metabolites), not explicitly mentioned, the regulatory limit of 0.1 µg/L applies. In groundwater, the regulatory limit is also fixed at 0.1 µg/L (Directive 2006/118/EC).

Monitoring data analysis was performed using the information technology system (Pesticide Monitoring Information System—SIMP) developed by ISPRA [15]. The system allows the acquisition, processing, and dissemination of information. The imported master data on the monitoring stations and the analytical determinations underwent a preliminary quality control and validation before processing. The system produces statistical outputs (tables, graphs) and spatial contamination maps.

## 3. Results

### 3.1. List of PFAS Active Substances

In 2011, the Organization for Economic Co-operation and Development (OECD) reviewed the terminology of PFASs, providing a harmonized general definition to comprehensively reflect the universe of PFASs [20]. The definition is based on molecular structure and, given the high complexity and diversity of PFASs, it still allows their categorization by combining characterization traits with additional considerations.

Starting from the definition given by the OECD, different categories of PFASs are currently used in scientific literature to address specific working scopes. This approach has produced different datasets of PFAS pesticides [22,23,24] that are internally coherent and consistent. In this study, we refer to the PFAS definition adopted in the proposal for a universal restriction of PFASs (cited in Section 2.1) [18]. The aim of the restriction proposal is to drastically reduce the use of PFASs that are either persistent themselves or degrade to equally persistent PFASs. A limited number of specific PFAS subgroups, with combinations of key structural elements for which it can be expected that they will ultimately mineralize in the environment, are therefore excluded from the scope of the proposal.

In this study, PFAS pesticides are identified based on the restriction proposal definition, although pesticide active substances, but not co-formulants, are excluded from the scope of the proposal, as their use is subject to the authorization procedure under the Plant Protection Product (PPP) Regulation (Reg. (EC) 1107/2009). Forty-six PFAS active substances have been identified [19], 29 of which are currently approved under the PPP Regulation (Table 1) and therefore authorized for use within the European Union.

Among the substances currently authorized for use, many have been identified as Candidate for Substitution (CfSs) (Regulation (EU) 2022/1252), i.e., substances of particular concern that are authorized for a shorter period (7 years rather than 15) in the absence of less hazardous alternatives. Specifically, many PFAS pesticides are identified as CfSs because they meet at least two of the properties related to persistence, bioaccumulation, and toxicity (PBT). The renewal of authorization has not been approved for 17 of the 26 PFAS active substances listed in the table. None of the related plant protection products are currently marketed or applied in Italy, except for those whose authorizations were revoked in 2025 (Flufenacet, Metaflumizone, and Penthiopyrad) due to a planned stock-disposal and phase-out period of approximately one year.

For each substance, the harmonized hazard classification, according to the CLP regulation (Reg. (EC) 1727/2008), is reported in the table. Where it has not been determined (ND), the authorization procedure under the PPP Regulation considers the classification proposed by the applicant.

### 3.2. Use of PFAS Pesticides in Italy

**Figure 1:**
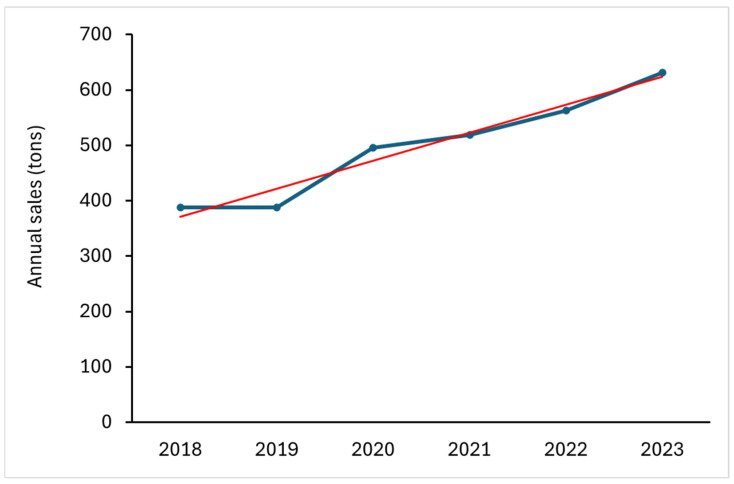
Sales in tons of the 46 PFAS pesticides in Italy (blue line) and trend line (red line). Source: ISTAT, Istituto Nazionale di Statistica (https://www.istat.it/en/).

Considering all active substances authorized in Europe [25], PFASs used as active substances represent over 8%, and their use is increasing. In Italy, annual sales data for all PFAS active substances show a 39% increase over the five-years period between 2018 and 2023 (Figure 1). In 2023, sales reached 632 tons.

The average sale volumes for each substance in 2023 is approximately 20 tons, however, for some substances, sale volumes are significantly higher. If average annual sales for substance is considered over the last three years, 2021–2023, the substances fluazinam and tefluthrin show the highest values, with over 100 tons sold, followed by lambda-cyhalothrin with approximately 40 tons, and by flufenacet and oxyfluorfen with volumes exceeding 30 tons.

### 3.3. Water Monitoring

The analytical measurements performed on water samples are compared with limits established by European and national laws. In the case of PFAS pesticides, regulatory concentration limits have not been established based on a toxicological assessment, but are instead generic limits set at 0.1 µg/L.

Monitoring data analysis was performed using an IT system (Pesticide Monitoring Information System—SIMP) developed by ISPRA [15]. This system represents a valuable tool for the acquisition, processing and dissemination of information.

**Figure 2:**
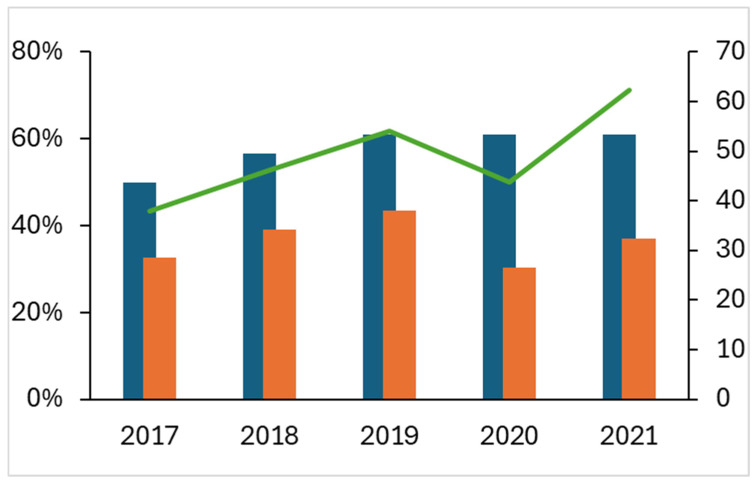
Percentage of monitored (blue bar) and detected (orange bar) PFAS pesticides compared to the total of 46 PFAS pesticides and number (×10^3^) of samplings (green line).

Over the five-year period from 2017 to 2021, 22–27 PFAS pesticides were monitored in surface water (rivers, lakes, transitional waters) and groundwater in Italy, representing 50–61% of 46 PFAS pesticides. The number of PFAS pesticides found in water varied between 14 and 20, representing 30–43% of 46 PFAS pesticides (Figure 2). These results are influenced by monitoring intensity, which depends both on the number of different substances investigated and the number of samples collected throughout the year. The lower number of samples in 2020 can be explained as a result of the restrictions imposed during the COVID-19 pandemic.

Table 2 lists the substances most frequently found during the five-year period. These are substances currently authorized for use; two of them, flufenacet and fluopicolide, are approved as Candidates for Substitution because they meet two of the three PBT criteria.

Table 2 shows the average frequency of detection in surface and groundwater during 2017–2021. Multiple factors influence the environmental fate of substances, including the physical–chemical properties of the molecules, but also to the hydrogeological characteristics of the geographic area. The organic carbon/water partition coefficient (Log Koc) can be correlated with mobility, such that low values correspond to a greater potential to reach groundwater through the soil. Average sales during 2021–2023 range between 6 and 32 tons, which correspond to low-to-medium-high values compared to the average sales of the total of 46 PFAS active substances.

## 4. Discussion

Based on the restriction proposal definition, 46 PFAS pesticides have been identified. However, the group of PFAS pesticides does not currently have a formally harmonized definition. A survey conducted by the European Food and Safety Authority (EFSA) identifies a non-exhaustive list of 140 PFAS pesticides [26], less than half of which have undergone peer review or are under review for PPP authorization. In 2023, the European Commission requested the EFSA to explicitly indicate in its conclusions which pesticide active substances and metabolites qualify as PFASs based on their chemical structure, in line with the REACH restriction proposal [26].

PFASs used as active substances in PPPs are generally characterized by the presence of one or more CF_3_-group(s) in their molecular structure that modify properties such as stability and lipophilicity, providing increased bioavailability [27]. Correspondingly, there has been an increase in sales in Europe: in the Netherlands between 2020 to 2022, sales increased by 83.2%, rising 250 tons [28]; sales in France in 2021 amount to more than 2000 tons [29]. PFAS pesticides in Europe are estimated at 5479 t/y, corresponding to approximately 2% of total PFASs used in Europe [19].

Sale volumes are related to the quantities used in agriculture, which involve the deliberate release into various environmental matrices, with the consequence of potential environmental contamination. PFAS pesticide residues in the environment from year to year will likely accumulate on residues released into the environment in previous years and which still persist, due to the typical properties of PFASs. Contamination of environmental matrices can be verified through environmental monitoring. Pesticide monitoring in surface and groundwater is planned based on an assessment of the environmental impact of their use. The risk of water contamination primarily accounts for pesticide use, their hazardous nature, and temporal trends observed in monitoring results over the years. In addition to the environmental pressure exerted by the active substances, it is necessary to consider the contribution of contaminants from co-formulants and any pesticide transformation products, for which information is often lacking. Moreover, the regulatory reference limit of 0.1 µg/L is likely inadequate for risk assessment, given that the hazardous properties of these substances could produce significant effects even at very low concentrations.

Physical–chemical properties influence the environmental fate of pesticides and their potential to reach groundwater by leaching through the soil. The preferential presence of some PFAS pesticides in groundwater is due to the high mobility typical of many PFASs ([17], chapter 1.1.4.4. Mobility), [30]. Indeed, the organic carbon/water partition coefficient (Log Koc) describes the tendency of a chemical to be adsorbed by the organic fraction present in the soil (or sediment). Higher Log Koc values are correlated with less mobile chemicals in soil, while lower Log Koc values correspond to more mobile chemicals. This correlation is evident among the most detected PFAS pesticides in Table 2. The active substance fluazifop-P, considered highly leachable [31], is the most frequently detected in groundwater, although it has the lowest average tonnage sold in 2021–2023. Flonicamid, with the lowest Log Koc value, is also among the most frequently detected compounds in groundwater. In contrast, the detection of cyflufenamid only in surface water could be attributed to its low mobility. The occurrence of Tetraconazole in groundwater is probably due primarily to its high average sales rather than its mobility.

The literature collected during the ongoing work on the universal restriction proposal [18] highlights that as the length of the perfluoroalkyl chain of PFASs decreases (<C8), their mobility and solubility in water increase, while their affinity for sediments and organic matter decreases. This behavior is mainly due to hydrophobic interactions of long-chain PFASs with organic matter and soil particles, while short-chain PFASs tend to leach into groundwater due to their higher solubility [32,33]. However, other factors influence the environmental fate of PFASs, such as the presence of functional groups and the ability to form hydrogen bonds and undergo coordination or complexation with soil minerals such as metal oxides and clays [34]. Soil pH further modulates PFAS retention in soil [35], as do salinity and the presence of competing anions [36]. The affinity for water can also vary as a result of environmental degradation processes (e.g., oxidation or hydrolysis). The environmental fate of PFASs is therefore influenced by several factors, making accurate prediction challenging.

One of the main degradation products of PFAS pesticides is trifluoroacetic acid (TFA). This very mobile PFAS does not undergo further degradation and, once released into the environment, persists and accumulates. Over the years, TFA has become one of the most widespread water contaminants [37,38], and PFAS pesticides represent a significant source of TFA [23,39]. PFAS monitoring programs in Italy have largely focused on long-chain compounds [40]. In contrast, the presence and distribution of ultra-short-chain PFASs (USC-PFASs) across Italy remain insufficiently characterized, with only limited data available for drinking water, surface water, and groundwater. A recent screening of TFA conducted in Central and Northern Italy [41] reports its occurrence in surface waters, mineral and spring waters, tap water, and even in both alcoholic and non-alcoholic beverages. Atmospheric deposition has been suggested as a potential source of this widespread contamination. However, studies investigating the origins of TFA in water, particularly those assessing contributions from different emission pathways, including agricultural uses, are still lacking.

TFA is a metabolite of flufenacet and flonicamid [31], which are among the PFAS pesticides most frequently detected at national level. In the Regulation on the non-renewal of the authorization of flufenacet (Regulation (EU) 2025/910), one of the grounds cited is the formation of the metabolite TFA. Indeed, TFA is indicated by EFSA as a substance that meets the criteria for classification as toxic for reproduction, category 2, pursuant to Regulation (EC) 1727/2008, as well as being a potential contaminant of groundwater, with levels that can substantially exceed the regulatory limit of 0.1 μg/L. Moreover, there is a proposal to classify TFA as Acute toxicity, category 3 (H331), Reproductive toxicity, category 1B (H360Df), persistent, mobile and toxic (PMT) (H450), and very persistent and very mobile (vPvM), (H451) according to CLP criteria [42].

Following a request from the European Commission, EFSA, in cooperation with ECHA, is currently reassessing the health-based reference values for TFA [43]. EFSA and ECHA have also been jointly mandated to evaluate the fate and behavior of TFA in soil and water [44]. The TFA leaching potential from pesticide applications could lead to concentrations of concern in groundwater, which may not be correctly estimated under current regulatory procedures. Standard studies used for the authorization of PPPs may not be effective in assessing TFA formation, due to their limited duration when applied to persistent active substances [45]. The proposed universal restriction aims to eliminate or minimize the use also of precursor substances that lead to the formation of highly persistent degradation products. Inconsistent approaches to risk management for these substances, such as in the case of the approval of PFAS pesticides from which persistent metabolites may originate, may delay the transition to safer alternatives and reduce incentives for the development and adoption of less persistent active substances.

The common characteristic of the PFASs subject to the proposed restriction is their very high persistence. This property, together with additional concerns, is considered sufficient to justify a ban on the use of PFASs that are either persistent themselves or degrade to persistent PFASs. Persistence, on the other hand, is not the primary parameter guiding the pesticide authorization process. The PPP Regulation (Reg. (EC) 1107/2009) requires that the authorization to place a plant protection product on the market be preceded by a thorough risk assessment covering both health and environmental aspects. Risk assessment involves examining the distribution patterns of active substances and their degradation products in the different environmental compartments, as well as an assessment of the damage that such preparations may cause to human and animal health or any unacceptable effects on the environment. Predictive exposure models are used to determine expected concentrations in groundwater and surface water. Therefore, the risk associated with each substance is characterized by calculating the ratio between short- and long-term toxicity and the expected exposure concentration. It is acknowledged that the risk assessment under the PPP Regulation ensures a high level of safety for the protection of the environment and human health; however, it does not specifically address the concerns arising from PFAS properties as foreseen in the restriction proposal under the REACH regulation.

A general review of the authorization approach would therefore be desirable, particularly with regard to predictive models for the concentration of active substances and their metabolites. The application of the current risk-based approval approach of PFAS pesticides would lead to the accumulation of these contaminants in the environment and to their wider territorial diffusion, thereby undermining the European objectives of a pollutant-free environment.

The direct application of PFAS pesticides on agricultural soils increases the likelihood of migration into surface and groundwater, resulting in greater challenges for water resource remediation aimed at meeting drinking water standards. Different assessment methodologies and regulatory thresholds across sectors continue to apply, demonstrating that harmonization through the “One Substance One Assessment” approach has not yet been fully achieved.

## 5. Conclusions

The results reported in this work provide an initial insight into PFAS subgroup that is intentionally widely released directly into the environment for plant protection purposes. Despite existing EU regulatory frameworks, the use of PFAS pesticides leads to measurable and widespread water contamination in Italy, a problem that is likely underestimated by current monitoring strategies due to the limited number of active substances tested, as well as the lack of investigation of PFAS metabolites and co-formulants.

Given their concerning properties-including high persistence, bioaccumulation and/or mobility in environmental matrices, and (eco-)toxicological effects-together with the significant water contamination potential and limited capacity for environmental remediation, a drastic and timely reduction or elimination of PFAS pesticide release could represent the most effective solution. Such an approach would be consistent with the objectives of the proposed universal PFAS restriction and, more broadly, with the aim of the European Strategies on chemicals. In the context of the EU’s “One Substance One Assessment” approach, it would therefore be appropriate to reflect on the need to address the same concerns in a consistent manner across different regulatory frameworks.

## Figures and Tables

**Table 1:** PFAS active substances identified based on the definition provided in the PFAS restriction proposal: approval status, harmonized classification, and function.

PFAS Active Substances	Approval Status in 2026		Harmonized Classification ^1^	Function
	Approval Review Date	Type of Approval		
Acrinathrin	Not approved		ND ^2^	acaricide/insecticide
Beflubutamid	31 October 2026		Aquatic Chronic 1 H410	herbicide
Benfluralin	Not approved		Carc. 2 H351; Repr. 2 H361d; Skin Irrit. 2 H315; Eye Irrit. 2 H319; Skin Sens. 1 H317; Aquatic Acute 1 H400; Aquatic Chronic 1 H410	herbicide
Bifenthrin	Not approved		Carc. 2 H351; Acute Tox. 2 (Oral) H300; Acute Tox. 3 (Inhalation) H331; STOT RE 1 H372; Skin Sens. 1B H317; Aquatic Acute 1 H400; Aquatic Chronic 1 H410	acaricide/insecticide
Cyflufenamid	30 June 2027		ND	fungicide
Cyflumetofen	15 October 2027		Carc. 2 H351; Skin Sens. 1 H317	acaricide
Diflufenican	31 August 2027	CfS	Aquatic Acute 1 H400; Aquatic Chronic 1 H410	herbicide
Fipronil	Not approved		Acute Tox. 3 H301, Acute Tox. 3 H311, Acute Tox. 3 H331, Aquatic Acute 1 H400, Aquatic Chronic 1 H410, STOT RE 1 H372	insecticide
Flazasulfuron	31 July 2032		Aquatic Acute 1 H400; Aquatic Chronic 1 H410	herbicide
Flonicamid	30 November 2026		Acute Tox. 4 (Oral) H302	insecticide
Fluazifop-P	31 May 2026		ND	herbicide
Fluazinam	15 April 2026		Repr. 2 H361d; Eye Dam. 1 H318; Acute Tox. 4 (Inhalation) H332; Skin Sens. 1 H317; Aquatic Acute 1 H400; Aquatic Chronic 1 H410	fungicide
Flubendiamide	Not approved		ND	insecticide
Flufenacet	Not approved		Acute Tox. 4 (Oral) * H302; STOT RE 2 * H373 **; Skin Sens. 1 H317; Aquatic Acute 1 H400; Aquatic Chronic 1 H410	herbicide
Flufenoxuron	Not approved		Aquatic Acute 1 H400, Aquatic Chronic 1 H410, Lact. H362	insecticide
Flumetralin	Not approved		Skin Irrit. 2 H315; Eye Irrit. 2 H319; Skin Sens. 1 H317; Aquatic Acute 1 H400; Aquatic Chronic 1 H410	herbicide
Fluometuron	15 July 2026	CfS	ND	herbicide
Fluopicolide	31 August 2026	CfS	Repr. 2 H361d	herbicide
Fluopyram	30 June 2026		Aquatic Chronic 2 H411	fungicide
Flurochloridone	31 October 2027	CfS	Repr. 1B H360FD; Skin Sens. 1 H317; Acute Tox. 4 (Oral) H302; Aquatic Acute 1 H400; Aquatic Chronic 1 H410	herbicide
Flutianil	14 April 2029		Aquatic Chronic 1 H410	fungicide
Flutolanil	15 June 2026		ND	fungicide
Gamma-Cyhalothrin	Not approved		ND	insecticide
Haloxyfop-P	Not approved		ND	herbicide
Isoxaflutole	31 July 2034		Repr. 2 H361d ***; Aquatic Acute 1 H400; Aquatic Chronic 1 H410	herbicide
Lambda-Cyhalothrin	31 August 2026	CfS	Acute Tox. 2 (Inhalation) * H330; Acute Tox. 4 (Dermal) * H312; Acute Tox. 3 (Oral) * H301; Aquatic Acute 1 H400; Aquatic Chronic 1 H410	insecticide
Mefentrifluconazole	20 March 2029		Skin Sens. 1 H317; Aquatic Acute 1 H400; Aquatic Chronic 1 H410	fungicide
Metaflumizone	Not approved		Repr. 2 H361fd; Lact. H362; STOT RE 2 H373	insecticide
Oxathiapiprolin	3 March 2027		Aquatic Chronic 1 H410	fungicide
Oxyfluorfen	31 May 2027	CfS	ND	herbicide
Penoxsulam	15 May 2026		ND	herbicide
Penthiopyrad	Not approved		ND	fungicide
Picolinafen	30 June 2031		STOT RE 2 H373; Aquatic Acute 1 H400; Aquatic Chronic 1 H410	herbicide
Picoxystrobin	Not approved		ND	fungicide
Prosulfuron	15 June 2026	CfS	Acute Tox. 4 (Oral) * H302; Aquatic Acute 1 H400; Aquatic Chronic 1 H410	herbicide
Pyridalyl	Not approved		Skin Sens. 1 H317; Aquatic Acute 1 H400; Aquatic Chronic 1 H410	insecticide
Pyroxsulam	30 September 2027		Skin Sens. 1 H317; Aquatic Acute 1 H400; Aquatic Chronic 1 H410	herbicide
Sulfoxaflor	18 January 2028		Acute Tox. 4 (Oral) H302; Aquatic Acute 1 H400; Aquatic Chronic 1 H410	insecticide
Tau-Fluvalinate	31 January 2027		Acute Tox. 4 (Oral) * H302; Skin Irrit. 2 H315; Aquatic Acute 1 H400; Aquatic Chronic 1 H410	insecticide
Tefluthrin	31 May 2027		Acute Tox. 1 (Inhalation) H330; Acute Tox. 2 (Dermal) H310; Acute Tox. 2 (Oral) H300; Aquatic Acute 1 H400; Aquatic Chronic 1 H410	insecticide
Tembotrione	31 December 2026	CfS	Repr. 2 H361d; STOT RE 2 H373; Skin Sens. 1 H317; Aquatic Acute 1 H400; Aquatic Chronic 1 H410	herbicide
Tetraconazole	31 March 2027		Acute Tox. 4 (Inhalation) * H332; Acute Tox. 4 (Oral) * H302; Aquatic Chronic 2 H411	fungicide
Trifloxystrobin	31 July 2033		Lact. H362; Skin Sens. 1 H317; Aquatic Acute 1 H400; Aquatic Chronic 1 H410	fungicide
Triflumizole	Not approved		Acute Tox. 4 H302; Aquatic Acute 1 H400; Aquatic Chronic 1 H410; Repr. 1B H360D; STOT RE 2 H373; Skin Sens. 1 H317	fungicide
Triflusulfuron-methyl	Not approved		Carc. 2 H351; Aquatic Acute 1 H400; Aquatic Chronic 1 H410	herbicide
Tritosulfuron	Not approved		Skin Sens. 1 H317, Aquatic Acute 1 H400, Aquatic Chronic 1 H410	herbicide

^1^ For the meaning of the Hazard classes codes, please see the following link: https://echa.europa.eu/clp (accessed on 15 February 2026). ^2^ ND: not determined. * Minimum classification. ** Route of exposure cannot be excluded. *** Hazard statements for reproductive toxicity.

**Table 2:** PFAS pesticides most frequently detected in the period 2017–2021.

PFAS Active Substances	Average Frequency of Detection		Log Koc ^1^	Average Sales in 2021–2023 (tons)
	Surface Water	Groundwater		
Cyflufenamid	4.2	0.0	3.2	10.5
Flonicamid	5.3	10.8	0.8	13.7
Fluazifop-P	0.7	2.7	1.7	6.3
Flufenacet ^2^	2.1	0.2	2.3	32.0
Fluopicolide ^2^	3.6	0.2	2.5	8.8
Tetraconazole	3.6	3.1	3.0	28.5

^1^ Koc: organic carbon/water partition coefficient, source: https://www.sifdataweb.it. ^2^ Identified as Candidates for Substitution.

## Data Availability

The data presented in this study are openly available in ISPRA-Portale Pesticidi at https://sinacloud.isprambiente.it/portal/apps/sites/#/portalepesticidi, (accessed on 25 February 2026) reference number [14]. [ISPRA, Portale Pesticidi] [https://sinacloud.isprambiente.it/portal/apps/sites/#/portalepesticidi] [15].
